# Implementation of ISO/IEEE 11073 PHD SpO2 and ECG Device Specializations over Bluetooth HDP following Health Care Profile for Smart Living

**DOI:** 10.3390/s22155648

**Published:** 2022-07-28

**Authors:** Alexandra Cristobal-Huerta, Angel Torrado-Carvajal, Cristina Rodriguez-Sanchez, Juan Antonio Hernandez-Tamames, Maria Luaces, Susana Borromeo

**Affiliations:** 1 Electronic Technology Area, Universidad Rey Juan Carlos, 28933 Madrid, Spain; a.cristobalhuerta@gmail.com (A.C.-H.); angel.torrado@urjc.es (A.T.-C.); cristina.rodriguez.sanchez@urjc.es (C.R.-S.); juan.tamames@urjc.es (J.A.H.-T.); 2 Medical Image Analysis and Biometry Lab, Universidad Rey Juan Carlos, 28933 Madrid, Spain; 3 Hospital Universitario Clínico San Carlos, 28040 Madrid, Spain; mluaces@salud.madrid.org

**Keywords:** Bluetooth HDP, Continua Health Alliance guidelines, digital signal processors (DSPs), electrocardiography, ISO/IEEE standards, SpO2, telemedicine

## Abstract

Current m-Health scenarios in the smart living era, as the interpretation of the smart city at each person’s level, present several challenges associated with interoperability between different clinical devices and applications. The Continua Health Alliance establishes design guidelines to standardize application communication to guarantee interoperability among medical devices. In this paper, we describe the implementation of two IEEE agents for oxygen saturation level (SpO2) measurements and electrocardiogram (ECG) data acquisition, respectively, and a smartphone IEEE manager for validation. We developed both IEEE agents over the Bluetooth Health Device Profile following the Continua guidelines and they are part of a telemonitoring system. This system was evaluated in a sample composed of 10 volunteers (mean age 29.8 ± 7.1 y/o; 5 females) under supervision of an expert cardiologist. The evaluation consisted of measuring the SpO2 and ECG signal sitting and at rest, before and after exercising for 15 min. Physiological measurements were assessed and compared against commercial devices, and our expert physician did not find any relevant differences in the ECG signal. Additionally, the system was assessed when acquiring and processing different heart rate data to prove that warnings were generated when the heart rate was under/above the thresholds for bradycardia and tachycardia, respectively.

## 1. Introduction

M-Health was first introduced as “Unwired e-med” in the *IEEE Transactions on Information Technology in Biomedicine* journal in 2000 [1]. Over the past 20 years, the development and advancements in information and communication technologies (ICTs) and connectivity has impacted m-Health directly. In this sense, smart living is the interpretation of the smart city but at the level of each person’s home, creating a new lifestyle thanks to the inclusion of these ICT improvements in their own personal spaces. This new way of living involves, among others, the fields of health, safety, home automation, and smart services, where new technologies are present in the complete cycle of patient treatment. Concretely, new scenarios have emerged where healthcare professionals have access to the Hospital Information System (HIS) anywhere and anytime [2,3].

In this framework, new technologies should be present in the complete cycle of patient treatment and ICTs can improve healthcare in developed economies by minimizing unnecessary visits to a hospital [4,5,6]. These techniques and devices for early measurement of parameters allow for the continuous and ubiquitous monitoring of patients through medical decision support applications (alarms, shared diagnosis, DSS (Decision Support systems), etc.), helping in the diagnosis, monitoring, and intervention of patients. The deployment of these applications improves the ubiquity of healthcare providers in hospitals, increasing the time they spend with patients [6,7,8,9,10]. However, the new paradigm of health care in urban environments brings with it the implementation of ICT-intensive devices with smaller, higher-performance electronic sensors, and systems with greater interactivity to improve health management. This requires the optimization of interoperable medical sensor networks with terminals to fasten responses in emergency situations where a rapid actuation could be crucial, impacting directly on the efficient use of the infrastructure and reducing costs related to clinical environments.

In this scenario, where many prototypes of m-health systems based on the Body Area Network (BAN) are becoming popular, the main drawback these systems face in the smart living approach is the lack of interoperability among medical devices [11], which could promote a homogeneous e-health ecosystem [12]. Furthermore, the current situation presents several challenges associated to the certification and regulation of these devices and their software to be interoperable [13]. Clinical applications must adapt to a paradigm in which results are repeatable and reliable. In this context, standards are crucial to the advancement of smart cities and smart living to help smooth the implementation of innovative technologies and deliver a reliable framework for practitioners. In addition, the interfaces must follow these standards to be integrated in a hospital infrastructure without many changes. Thus, the development and maintenance processes must be agile [14].

**Figure 1:**
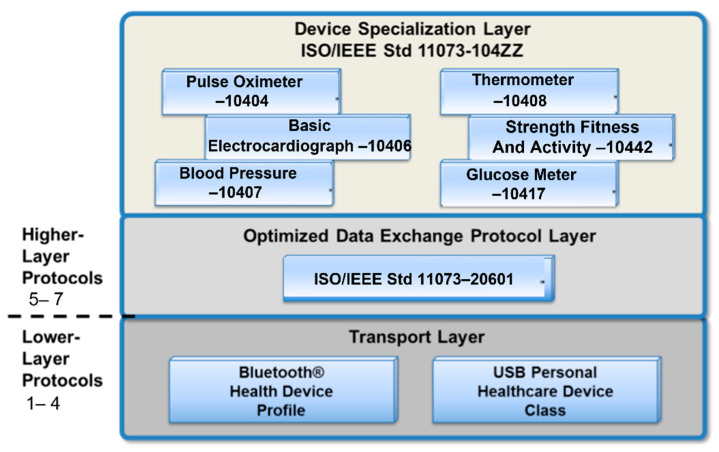
Organization and dependencies of the protocols established by the Continua Health Alliance, over the Open Systems Interconnection (OSI) model.

ISO/IEEE 11073 “Health informatics—Point-of-care medical device and Personal Health Device (PHD) communication standards” is a family of standards that enable communication between medical, healthcare, and wellness devices with external computer systems. These standards are based on a single framework, ISO/IEEE 11073-20601, and several device specializations (Figure 1). They provide automatic and detailed electronic data capture of client-related and vital signs information and of device operational data. The IEEE 11073 Personal Health Devices (PHD) initiative was designed to accommodate the capabilities of low-power embedded devices and very low-power wireless technologies. Additionally, the use of transport technologies was designed to adapt them to this interface, resulting in the base protocol IEEE 11073-20601. Bluetooth, USB, ZigBee, and NFC each developed a standard (most recently NFC), and these are profiled by the Continua Alliance guidelines to apply these standards and guarantee interoperability between medical devices. This methodology establishes a product certification program, as well as collaboration with government regulatory agencies.

### 1.1. Continua Health Alliance Design Guidelines

Continua Health Alliance’s design guidelines are focused on the Interface to Personal Area Network health devices (PAN-IF) and the Interface between Disease Management Services (DMS), Wireless Area Network (WAN) devices (xHR Senders), and Electronic Health Record (EHR) devices (xHR Receivers) (xHRN-IF). Continua follows the Open Systems Interconnection (OSI) model to establish the protocols to use for interoperability in the PAN-IF. The protocols used in the different OSI layers can be seen in Figure 1 [15].

Continua has constrained the lower-level protocol standards for Bluetooth and USB communications with the use of specific profiles for health devices. For the higher layers, Continua uses the ISO/IEEE 11073 standards family, concretely the IEEE 11073-20601-Optimized Exchange Protocol and ISO/IEEE 11073-104xx-device specializations to provide application-level interoperability.

The ISO/IEEE 11073 series of standards is based on an object-oriented system management paradigm. The system model is divided into three principal components: the domain information model (DIM), the service model, and the communication model.

The DIM describes an agent as a set of objects with attributes, which represent the behavior of the data that can be sent to the manager. The communication between the agent and the manager is defined by the ISO/IEEE 11073-20601 standard. The service model defines the messages that an agent and a manager interchange following Abstract Syntax Notation One (ASN.1). These messages are encoded in Medical Device Encoding Rules (MDER), described in the ISO/IEEE 11073-20101 standard. The communication model supports the topology of one or more agents communicating over logical point-to-point connections to a single manager. A connection state machine, specified in ISO/IEEE 11073-20601, defines the system behavior for each connection.

Above the IEEE 11073-20601 exchange protocol, are the device specializations that describe specific details about how a type of agent works and the objects and attributes that it has. For the system implemented in this paper, the pulse oximeter (IEEE 11073-10404) and the recently basic ECG (IEEE 11073-10406) specializations were used.

### 1.2. Our Contribution

The aim of this paper is to investigate the feasibility of the SpO2 and ECG medical devices, following the Continua guidelines [16], for increasing the adherence of patients to a cardiac rehabilitation treatment. Cardiac rehabilitation patients have cardiovascular problems. Vital signs such as heart rate (HR) and oxygen saturation are used as predictors of recovery after surgery or in patients with coronary artery disease (CAD) who underwent rehabilitation [17,18]. When they are being evaluated or undergo physical training, it is essential to monitor their blood pressure and heart rate (HR) both at rest and the increase during the exercise they are doing, measuring baseline blood pressure and heart rate and peak effort. In addition, SpO2 is measured because if it drops below 90%, training must be stopped. Therefore, the measure of SpO2 and HR is mandatory for any patient undergoing cardiac rehabilitation treatment or high-risk patients [19]. In the next sections, we describe our system architecture consisting of two modules. The hardware module includes a description of the signal acquisition, processing, and transmission, corresponding with the IEEE agent-node that transmits personal health data. The software module includes a description of an Android application that receives processed information, corresponding with the IEEE manager-node that receives personal health data. Finally, results of first clinical trials, a discussion, and conclusions are presented.

## 2. Materials and Methods

### 2.1. Requirements

**Medical Requirements**

SpO2 and ECG data collection are required in order to evaluate the adherence of patients to a cardiac rehabilitation treatment. Analysis of these biosignals may produce alarms to warn about emergency events. For SpO2, the oxygen saturation level must be higher than a minimum safety threshold (90–95% [18,19,20]). If the level decreases under this threshold, a warning must be generated.

For ECG, lead II was registered as it is the best ECG lead in order to record heart rhythm and heart rate [21]. The lead II has the configuration: II (RA—Right Arm; LA—Left Arm; and RL—Right Leg). Pediatric ECG electrodes, round fabric, 40 mm diameter, wet gel, snap electrode, silver chloride (Ag/AgCl) sensor, repositionable. Our clinicians wanted to track the changes in the heart rate (HR) such as bradycardia (HR < 60 beats per minute (bpm)) and tachycardia (>100 bpm) [22]. In the case of such an event, we must acquire and store at least 10 minutes of signal; this corresponds to 5 min before and 5 min after the warning event.

**Technical Requirements**

Real-time and wear specifications have resulted in a system with fixed latency, power supply, and space limitations. Medical requirements have led to the inclusion of storage capacities.

In this work, we proposed the use of a DSP as the processing element. As the architecture of a DSP is optimized specifically for digital signal processing, it is the best solution regarding the performance and cost in relation to others, such as FPGAs or microprocessors.

For clinical use, the signals should be digitalized with at least 12 bits of resolution. Moreover, several algorithms should be included to remove noise, extract parameters from physiological data, and identify warning events.

### 2.2. System Description

**Figure 2:**
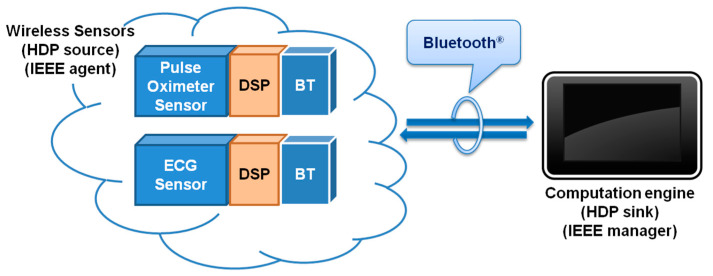
IEEE agent for physiological data acquisition, processing, and transmission (**left**); and IEEE manager smartphone application for patient empowerment (**right**).

Our system consists of two different modules corresponding with the two IEEE configuration modes over a Bluetooth connection using the Health Device Profile (HDP) (Figure 2). These modules were developed following a Service Oriented Architecture (SOA). Connectivity, semantic interoperability, and security are ensured using ISO/IEEE 11073 over Continua-certified communication modules. Material and methods are described below for each module.

#### 2.2.1. IEEE Agent

**Figure 3:**
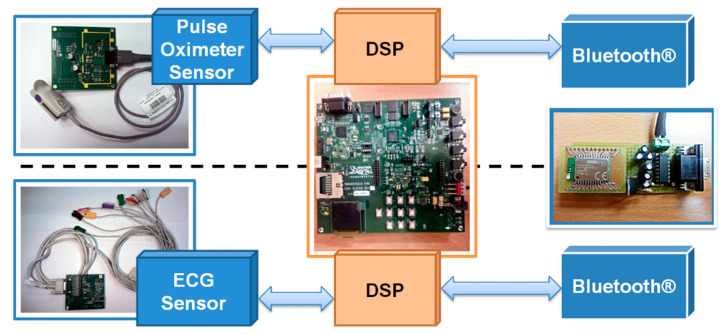
Architecture of the IEEE agent in our framework showing the acquisition, processing, and transmission hardware.

HDP defines a transmitter of medical data as a source; ISO/IEEE-11073-20601 uses the term IEEE agent for this same node. In our framework, this agent corresponds to the acquisition, processing, and transmission hardware (Figure 3) [23].

**Data Acquisition.** Our framework is composed of the TMDXMDKPO8328 and ADS1298RFE boards from Texas Instruments (TI) for SpO2 and ECG data acquisition. Both, the pulse oximeter and the ECG implementation, are provided for the TMS320C5515 DSP as part of the Medical Development Kit (MDK) intended for evaluation and development purposes.

The SpO2 acquisition board includes a low-power analog-to-digital converter (ADC), with 16 bits of resolution and 500 kilo samples per second (ksps) sampling rate. After acquiring the absorption value of the red (R) and infrared (IR) rays for the blood, the signals are conditioned by two stages of an amplifier.

In the case of the ECG, the acquisition board includes a low-power ADC, working at 500 sps data rate with 24 bit data resolution. Analog signals pass through a defibrillator protection (DP) circuit to protect the rest of the system. The board derives 8 out of 12 ECG leads using differential amplifiers. These leads are filtered by a low pass filter (LPF), which provides anti-aliasing and removes frequencies above 150 Hz with 60 dB attenuation and digitized. Due to our medical requirements (see Section 2.1), only lead II is analyzed by the system.

Both boards interface with the DSP module using the serial peripheral interface (SPI) and the Inter-Integrated Circuit (I2C) buses.

**Bluetooth Communications.** Communication between the agent and the manager is implemented using the ISO/IEEE-11073-20601 optimized exchange protocol over a Bluetooth stack with HDP. This protocol provides plug-and-play interoperability by using the IEEE-11073-20601.

According to the established requirements for this proposal, our system includes a Bluegiga WT12-A Bluetooth module. The Bluegiga WT12 is a fully integrated Bluetooth 2.1 + Enhanced Data Rate (EDR), class 2 module combining an antenna, Bluetooth radio, and an on-board iWRAP Bluetooth stack including the iWRAP 5.0.0 firmware, which provides an easy way to configure Bluetooth to work with HDP. This version of iWRAP includes the implementation of several IEEE agents, which leads to a transparent connection management and data packaging. Thus, allowing a lower-end host processor to be used and faster time to market. The agents implemented in this firmware were tested to pass Continua v.1.5 test cases, which also allows avoiding the certification process, thus, saving time. We used the standard configuration for a pulse oximeter agent implemented in the iWRAP firmware.

**Figure 4:**
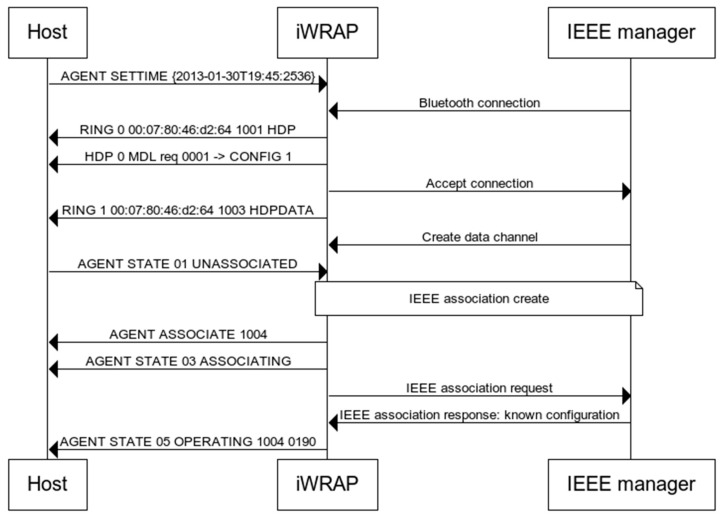
Example of connection establishment sequence between a manager and a SpO2 IEEE agent implemented in the iWRAP firmware. This figure shows the syntax and the order of the commands exchanged.

An example of a connection establishment sequence between a manager and a SpO2 agent implemented in the iWRAP firmware is shown in Figure 4.

Since the ECG agent is not supported by iWRAP, we developed an ECG agent with extended configuration in our system. It was explained in the ECG Data Processing subsection.

**Processing System.** Incoming data are processed by the low-power TMS320C5515 DSP Medical Development Kit (MDK) evaluation module (EVM) from TI. The implementation of the EVM only allows connecting one front-end-board at a time.

Our software implementations are based on the examples provided by TI. We also included Real Time Clock (RTC) and External Memory Interface (EMIF) peripherals from the C55x Chip Support Libraries (CSL). CSLs provide an application programming interface (API) that shortens development time by providing standardization and portability.

In the system, data are received from the acquisition boards through the SPI bus. They are processed to obtain the relevant information (plethysmographic waveform, SpO2 value, ECG waveform, and HR). Then, data are encapsulated following the corresponding IEEE device specialization standard. The most relevant algorithms and new characteristics included in our system are described in the following subsections.

*SpO2 Data Processing.* To acquire the SpO2 data, the DSP reads alternatively the R and IR values from SPI every 1 ms.

A first-order IIR filter is used to remove the DC component. Then, a Hamming windowed 51st order low-pass FIR filter removes unwanted frequencies above 10 Hz.

The SpO2 percentage value is obtained from the ratio of the Root Mean Square (RMS) of the R and IR signals using a look-up table (LUT) every three heart beats. The pulse rate is detected using the filtered IR signal. The values of samples from the plethysmographic waveform are calculated through a square root algorithm which provides a proportional value of the peak-to-peak signal. To provide a timestamp for the measure, we used the CSL to implement the RTC. Further details regarding the actual ECG implementation can be found in the TI Application Report SPRAB37A—June 2010 [24].

Once the SpO2 percentage value is calculated and its timestamp is obtained, the SpO2 value is compared with the threshold. Hence, if the mean SpO2 value is lower than 95%, the system generates an alarm. To avoid false alarms, we calculated the mean of the last 10 values of the SpO2 percentage. Moreover, the first four SpO2 values for each monitoring session were discarded to allow the signal to become steady.

In that moment, and if the connection between the agent and the manager is already established, the system sends the last value of SpO2 and the timestamp through the UART peripheral to the Bluetooth, following the iWRAP syntax (Table 1).

The UART peripheral is configured to work to 115,200 bauds, 8 data bits, no parity, and one stop bit. When the alarm has been sent, the system waits for the ACK from the manager.

Previously, to send an alarm, the agent and manager had to establish the connection following the state machine defined in IEEE 11073-20601. The state machine defines the states and substates an agent and manager pair pass through. The agent implementation in the iWRAP 5.0, corresponding to the standard SpO2, manages it.

A successful IEEE association is indicated to the DSP by an event message from the Bluetooth chip, and the agent will pass to the operating state. The message contains the channel and configuration identifiers of the IEEE configuration that the manager accepted. Then, the agent is ready to send data to the manager. If this state is not reached, the association with the manager has failed and the agent will pass to the unassociated state.

*ECG Data Processing.* An interruption is generated every 2 ms in order to read ECG data from the SPI bus. The DSP digitizes nine channels with 24 bits of resolution. The first channel is the lead-off status. The eight remaining channels are passed through the same IIR filter as the SpO2 signal, to remove the DC component. Removing unwanted frequencies is achieved using a multi-band-pass filter, which consists of a FIR filter and notch filter at 50/60 Hz. Once the data is filtered, the DSP computes the remaining four leads. Then, a QRS detection algorithm is used to detect the R-peak and compute the HR. The QRS algorithm is applied over the lead II and consists of a variable threshold method [25]. The HR is calculated when five R-peaks have been detected. Further details regarding the actual ECG implementation can be found in the TI Application Report SPRAB36A—July 2009 [26].

To accomplish the medical requirements, data from the previous five minutes are stored in an external memory to assess for potential current events. For this purpose, we used the 64 Mb mobile–SDRAM memory (MT48H8M16LF by Micron). We checked the HR value to store the data; if it was non-zero, we stored it, as well as the lead status, the timestamp, the previous five R-R intervals, and the ECG sample related to the lead II.

**Figure 5:**
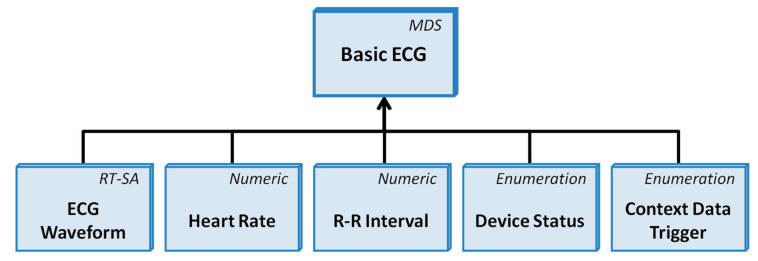
Representation of the different object classes in the ISO/IEEE 11073-10406 Basic ECG device specialization.

Based on the analysis of these data, the system decides if constants are out of range and an alarm must be generated. Given this situation, and given that devices are not already paired, the communication is established in a similar way as in the case of the SpO2 system, except that the DSP has to implement the state machine for the connection for the ECG. In this case, two data channels are opened, one reliable (for the main information) and one streaming (for the waveform samples). At that time, the system reads data from the SDRAM (from 5 min before the alarm if it is produced later than 5 min, or from the start of the monitoring session if it is produced before a 5 min monitoring session). The system reads all the values between two stored HR values. Then, it combines the data packet to accomplish the 11073-10406 standard. The system implements the object classes with mandatory and recommended attributes included in the specialization (Figure 5). One data packet is sent with HR, lead-off status, five R-R intervals in ms, and the timestamp through the reliable data channel.

After that, packets with 20 samples from lead II are sent through the streaming data channel until there are 20 or fewer samples to be sent. When this happens, the DSP reads from the SDRAM as before and sends a new data packet. In the same way as in the SpO2 system, data are sent through the UART (similarly configured). While the DSP is sending packets, it is acquiring and storing new data.

#### 2.2.2. IEEE Manager

In order to receive and visualize the processed data, a smartphone application was developed. The MORFEO OpenHealth Project developed the HDP/MCAP implementation on the official Linux Bluetooth protocol stack, BlueZ 4.77 [27]. This implementation was integrated by Google in Android API 27 and newer versions to support HDP through the Bluetooth Health class.

We used this class to implement an IEEE manager that is able to understand our SpO2 and ECG data through HDP.

When the HDP connection is established, the manager will wait for the agent application layer (Continua) association request. In this association, the agent and the manager establish the configuration and the manager starts receiving data.

**Figure 6:**
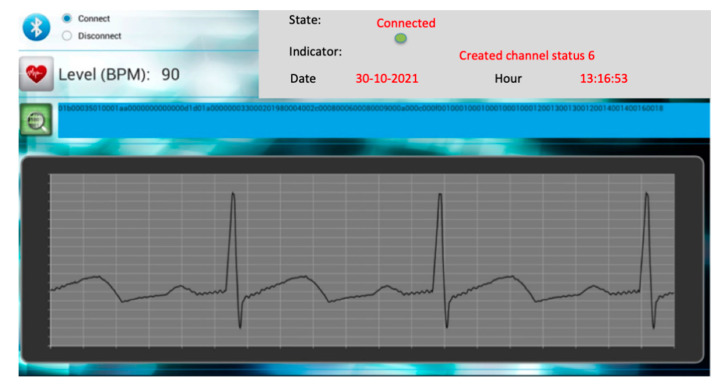
Graphical user interface of the ECG Android application, in which the Bluetooth connection status, the date and hour, heart rate, and ECG waveform are shown.

The application consists of a main menu that leads to two different modes, one for SpO2 and the other for ECG. In the SpO2 mode, the application provides a visualization of the connection status, the SpO2 level, and the date and hour of the sent packet. In the same way, in the ECG mode the application provides the visualization of the connection status, the HR, the date and hour of the acquisition and the ECG waveform. Figure 6 shows an example of the ECG application where the waveform from an ECG waveform generator is displayed. Although the evaluation versions included two screens with ECG and Sp02 data, the clinicians’ recommendation was to simplify with the ECG reading only.

### 2.3. Validation Methodology

The system was evaluated in a sample composed of 10 healthy volunteers (mean age 29.8 ± 7.1 y/o; 5 females) under supervision of an expert cardiologist from the Hospital Universitario de Fuenlabrada (HUF), Madrid, Spain. The research protocol was approved by the local Institutional Review Board and written informed consent was acquired from all participants. The evaluation consisted of measuring the SpO2 and ECG signals sitting and at rest, before and after exercising for 15 min. A 3-lead configuration was followed for the ECG recording with one electrode adjacent each clavicle bone on the upper chest (i.e., the left arm electrode (L lead) and the right arm electrode (R lead)), and a third electrode adjacent the patient’s lower left abdomen (i.e., the left leg electrode (N lead)).

Physiological measurements were assessed and compared against commercial devices. Warning events generated were monitored and received in a local server simulating the telemonitoring system in the hospital. To check the accuracy of the SpO2 measurement obtained with our system, we compared it with a commercial Nonin Onyx 9500 device. In the same way, the accuracy of the ECG signal was assessed against a General Electric (GE) MAC 1200 ST.

## 3. Results

### 3.1. Continua Guidelines Compliance

The validation of the accomplishment of Continua Guidelines was carried out in a different way for each system.

The SpO2 system accomplishes the Continua guidelines due to the use of the agent implementation in the iWRAP5 firmware on the WT12 chip. The firmware iWRAP5 was designed and tested to meet the requirements of Continua version 1.5.

As the iWRAP 5.0 firmware does not implement an ECG agent, the validation of the accomplishment of Continua guidelines for our ECG system was checked by following the Design Guidelines. Moreover, we made more validations using our own manager application. Further validations will include performing the Continua certification process.

The Android application can understand both IEEE agents implemented. This application is configured to be used in a very simple way.

### 3.2. Functional Validation

As aforementioned, we compared the measured data with a commercial Nonin Onyx 9500 device (Sp02_b_) to check the accuracy of the SpO2 measurement obtained with our system (Sp02_a_). Both the error in accuracy (mean Sp02_a_–Sp02_b_ difference) and the error in precision (SD of differences) remained below 1%. This obtained error is within the error allowed according to medical requirements from the hospital.

In the same way, the accuracy of the ECG signal was compared against a General Electric (GE) MAC 1200 ST. Our expert physician did not find any relevant differences in the recorded ECG signal. The results of the physician’s evaluations were accepted for both SpO2 and ECG signals. Additionally, our system acquired and processed data from an ECG simulator at different HRs to prove that HR was properly identified and that warnings were generated when the HR was under or above the thresholds for bradycardia and tachycardia, respectively. Therefore, using devices and applications based on standards, as the one reported in this paper, would facilitate the use and integration of different measuring and mobile devices.

## 4. Discussion

In this paper, we described the implementation of two IEEE agents for SpO2 and ECG data acquisition, respectively, and an IEEE manager for validation. We developed both IEEE agents over Bluetooth HDP following the Continua guidelines. The proposed architecture is modular, meeting the wireless standards and allowing for real-time processing. Unlike application frameworks that require structural changes and create complex interdependencies, our model is service-oriented. This solution maintains a reliable, resilient, integrated, interoperable, and cost-effective system for personalized healthcare, while incorporating ubiquity. The modular design adopted is intended to provide a future-proofed system, whose functionality may be upgraded by modifying the hardware or software. The system features include automatic or on-demand data recording mode and warning state detection, such as abnormal oxygen levels, bradycardia, or tachycardia. Nevertheless, because of the modular nature of our implementation and the flexibility of the standard, new algorithms such as ventricular extrasystole detection algorithms could be implemented in the IEEE agents, and the warning events generated by such new algorithms could be sent over the same protocol implementation.

Our expert physician checked the reliability of SpO2 and ECG signals captured by the system, and reported that both SpO2 values and ECG tracings provided by our system are reliable and acquired data are feasible for clinical use as this type of information is currently required in routine clinical practice. In this sense, we hypothesize that this system could be especially useful in cardiac rehabilitation programs, where a hard control of the health of the patient is needed to improve adherence. Actually, it has been shown that cardiac rehabilitation can reduce the morbimortality by practically 50% in patients with heart disease. Nevertheless, participation and adherence of patients to cardiac rehabilitation and its associated treatment remains low, especially among the elderly, women, and low socioeconomic profile patients [28]. In this scenario, the use of smartphone-based applications has proved an improvement in patient adherence to the treatment; for instance, Yudi et al. [29] showed that the use of smartphone-based rehabilitation led to a significantly higher uptake (94% vs. 68%, *p* < 0.05) and completion (80% vs. 47%, *p* < 0.05) of the program. Using devices and applications based on standards, such as the one reported in this paper, would facilitate the use and integration of different measuring and mobile devices.

## 5. Conclusions

In this paper, we described the implementation of two IEEE agents for SpO2 and ECG data acquisition, and an IEEE manager for validation. We developed both IEEE agents over Bluetooth HDP following the Continua guidelines. In particular, as far as we know, this implementation of the extended ECG agent is one of the first available for devices oriented to m-Health systems. These implementations offer a standardization of medical devices.

The system features include automatic or on-demand data recording mode and warning state detection, such as abnormal oxygen levels or changes in the HR such as bradycardia and tachycardia. Our expert physician checked the reliability of SpO2 and ECG signals digitized by the system. Due to this, this system can be useful in rehabilitation programs, when a hard control of the health of the patient is needed to improve adherence in cardiac rehabilitation.

Future work may improve the current system implementation for concurrent acquisition from different boards. For example, pulse oximeters are often used for estimating heart rate, obtaining the heart rate from the plethysmography signal; thus, the SpO2 agent could be extended to include this parameter; thus, we would have several sources to account for the HR. Additionally, new devices could include more personal health devices such as weighing scales, respiration rate, insulin pumps, medication monitors, etc. In particular, as far as we know, this implementation of the extended ECG agent is one of the first available implementations for devices oriented to m-Health systems. Finally, the objective of this work was the implementation of ISO/IEEE 11073 PHD SpO2 and ECG device specializations over Bluetooth HDP following the Health Care Profile. A qualitative and quantitative evaluation could be interesting in the methodology for our future work following recommendations from other authors [30,31,32].

## Figures and Tables

**Table 1:** Example of iWrap syntax to send pulse oximeter data.

Command	Description
AGENT UPDATE FFFF 1 A4C 97E0	Oxygen saturation is 97%
AGENT UPDATE FFFF 1 990 2013-01-30T20:05:2678	Time stamp of the measurement
AGENT UPDATE FFFF A A4C 74E0	Pulse rate is 74 bpm

## Data Availability

Not applicable.

## References

[1] Istepanian R.S.H., Laxminaryan S. (2000). UNWIRED, the next generation of wireless and interpretable telemedicine systems-editorial paper. IEEE Trans. Inform. Technol. Biomed..

[2] Kyriacou E.C., Pattichis C.S., Pattichis M.S. An overview of recent health care support systems for eEmergency and mHealth applications. Proceedings of the 2009 Annual International Conference of the IEEE Engineering in Medicine and Biology Society.

[3] Castellano N.N., Gazquez J.A., Salvador R.M.G., Gracia-Escudero A., Fernandez-Ros M., Manzano-Agugliaro F. (2015). Design of a real-time emergency telemedicine system for remote medical diagnosis. Biosyst. Eng..

[4] Rathbone A.L., Prescott J. (2017). The use of mobile apps and SMS messaging as physical and mental health interventions: Systematic review. J. Med. Internet Res..

[5] Tomasic I., Tomasic N., Trobec R., Krpan M., Kelava T. (2018). Continuous remote monitoring of COPD patients—justification and explanation of the requirements and a survey of the available technologies. Med. Biol. Eng. Comput..

[6] Santos D.F., Almeida H.O., Perkusich A. (2015). A personal connected health system for the Internet of Things based on the Constrained Application Protocol. Comput. Electr. Eng..

[7] Dwivedi A., Bali R.K., James A.E., Naguib R.N.G., Johnston D. Merger of knowledge management and information technology in healthcare: Opportunities and challenges. Proceedings of the IEEE CCECE2002. Canadian Conference on Electrical and Computer Engineering. Conference Proceedings (Cat. No.02CH37373).

[8] Mertz L. (2012). Ultrasound? Fetal Monitoring? Spectrometer? There′s an App for That!: Biomedical Smart Phone Apps Are Taking Healthcare by Storm. IEEE Pulse.

[9] Rodriguez-Sanchez M.C., Torrado-Carvajal A., Borromeo S., Hernandez-Tamames J.A., Luaces M. (2012). Novel Applications for M-Health and Free Messaging. IEEE Pervasive Comput..

[10] Kuroda T., Sasaki H., Suenaga T., Masuda Y., Yasumuro Y., Hori K., Ohboshi N., Takemura T., Chihara K., Yoshihara H. (2012). Embedded Ubiquitous Services on Hospital Information Systems. IEEE Trans. Inf. Technol. Biomed..

[11] Sevin A., Bayilmis C., Kirbas I. (2016). Design and implementation of a new quality of service-aware cross-layer medium access protocol for wireless body area networks. Comput. Electr. Eng..

[12] Lemlouma T., Laborie S., Rachedi A., Santos A., Vasilakos A.V. (2019). Special Issue on Selected Papers from e-Health Pervasive Wireless Applications and Services 2017. Information.

[13] Kumari A., Tanwar S., Tyagi S., Kumar N. (2018). Fog computing for Healthcare 4.0 environment: Opportunities and challenges. Comput. Electr. Eng..

[14] Rezaei F., Hempel M., Sharif H. (2018). A survey of recent trends in wireless communication standards, routing protocols, and energy harvesting techniques in E-health applications. Wearable Technologies: Concepts, Methodologies, Tools, and Applications.

[15] American Thoracic Society, American College of Chest Physicians (2003). ATS/ACCP Statement on cardiopulmonary exercise testing. Am. J. Respir Crit. Care Med..

[16] Carroll R., Cnossen R., Schnell M., Simons D. (2007). Continua: An interoperable personal healthcare ecosystem. IEEE Pervasive Comput..

[17] Canet J., Gallart L. (2013). Predicting postoperative pulmonary complications in the general population. Curr. Opin. Anaesthesiol..

[18] Evrengul H., Tanriverdi H., Kose S., Amasyali B., Kilic A., Celik T., Turhan H. (2006). The relationship between heart rate recovery and heart rate vari- ability in coronary artery disease. Ann. Noninvasive. Electrocardiol..

[19] Ambrosetti M., Abreu A., Corrà U., Davos C.H., Hansen D., Frederix I., Iliou M.C., Pedretti R.F.E., Schmid J.-P., Vigorito C. (2020). Secondary prevention through comprehensive cardiovascular rehabilitation: From knowledge to implementation. 2020 update. A position paper from the Secondary Prevention and Rehabilitation Section of the European Association of Preventive Cardiology. Eur. J. Prev. Cardiol..

[20] Understanding Pulse Oximetry SpO2 Concepts. Philips Medical Systems.

[21] Meek S., Morris F. (2002). ABC of clinical electrocardiography: Introduction. I—Leads, rate, rhythm, and cardiac axis. BMJ: Br. Med. J..

[22] Hampton J.R. (2008). The ECG Made Easy.

[23] (2009). Health Device Profile Implementation Guidance Whitepaper Medical Devices Working Group; Bluetooth Special Interest Group. https://www.bluetooth.com/wp-content/uploads/2019/03/HDP-Implementation_WP_V10.pdf.

[24] Markandey V. Pulse oximeter implementation on the TMS320C5515 DSP medical development kit (MDK). Texas Instruments Application Report Jun-2010. https://www.ti.com/lit/an/sprab37a/sprab37a.pdf?ts=1658835461222&ref_url=https%253A%252F%252Fwww.google.com%252F.

[25] Hamilton P.S., Tompkins W. (1986). Quantitative investigation of QRS detection rules using the MIT/BIH arrhythmia database. IEEE Trans. Biomed. Eng..

[26] Markandey V. ECG Implementation on the TMS320C5515 DSP Medical Development Kit (MDK). Texas Instruments Application Report Jul-2009. https://www.itfind.or.kr/COMIN/file29190-Pulse%20Oximeter%20Implementation%20on%20the%20TMS320C5515%20DSP%20Medical%20Development%20Kit%20MDK.pdf.

[27] Barrón-González H.G., Martínez-Espronceda M., Trigo J.D., Led S., Serrano L. (2015). Recent Advances in mHealth: An Update to Personal Health Device Interoperability Based on ISO/IEEE11073. Mobile Health.

[28] García-Bravo S., Cuesta-Gómez A., Campuzano-Ruiz R., López-Navas M.J., Domínguez-Paniagua J., Araújo-Narváez A., Barreñada-Copete E., García-Bravo C., Flórez-García M.T., Botas-Rodríguez J. (2021). Virtual reality and video games in cardiac rehabilitation programs. A systematic review. Disabil. Rehabil..

[29] Yudi M.B., Clark D.J., Tsang D., Jelinek M., Kalten K., Joshi S., Phan K., Nasis A., Amerena J., Arunothayaraj S. (2016). SMARTphone-based, early cardiac REHABilitation in patients with acute coronary syndromes [SMART-REHAB Trial]: A randomized controlled trial protocol. BMC Cardiovasc. Disord..

[30] Bland J.M., Altman D.G. (1999). Measuring agreement in method comparison studies. Stat. Methods Med. Res..

[31] Guber A., Gali E.S., Sarah K., David S. (2019). Wrist-Sensor Pulse Oximeter Enables Prolonged Patient Monitoring in Chronic Lung Diseases. J. Med. Syst..

[32] Sarmento A., Carlo V., Stefania P., Carolina L., Alessandra S., Flavia N., Massimo M., Leonardi A., Ossola D., Rigoni A. (2018). Qualitative and Quantitative Evaluation of a New Wearable Device for ECG and Respiratory Holter Monitoring. Int. J. Cardiol..

